# A Mobile Application for Direct Light Compensation in Smartphone-Based Fruit Image Acquisition Systems

**DOI:** 10.3390/s26134102

**Published:** 2026-06-28

**Authors:** Bruno Bernardi, Matteo Sbaglia, Giuseppe Papuzzo

**Affiliations:** 1 Dipartimento di Agraria, Università Mediterranea di Reggio Calabria, Loc. Feo di Vito, 89122 Reggio Calabria, Italy; matteo.sbaglia@unirc.it; 2 ICAR-C.N.R., Via Pietro Bucci, 8-9 C, 87036 Rende, Cosenza, Italy; giuseppe.papuzzo@icar.cnr.it

**Keywords:** computer vision, bergamot, fruit calibration, citrus colour index, precision agriculture, non-destructive testing, ArUco markers

## Abstract

This research represents an advancement in smartphone-based image acquisition methodology, building upon a previous study to estimate the essential oil content of bergamot fruits in situ using a deep learning approach. To overcome an operational constraint due to a bulky portable dark box to standardise illumination, this study proposes a more versatile solution: a mobile application based on a colour card reference. By replacing physical shielding with digital compensation, the app functions as a local colourimetric sensor, enabling real-time correction of images acquired directly in the orchard, regardless of environmental variables such as direct sunlight or shadows. Workflow relies on an automated calibration procedure. Upon image acquisition, the application utilises ArUco Markers to autonomously detect and extract both the colour card and the fruit surface. The core of the innovation lies in the colour calibration algorithm based on RGB histogram matching logic, which calculates the precise chromatic transformation required to align the field data with the reference card data (acquired under controlled conditions). These calculated parameters are then dynamically mapped onto the fruit’s image. The final output is a normalised high-fidelity image, ready for the calculation of chromatic indices, such as the citrus colour index, or for seamless integration into predictive models. The results show that the application is a valid tool for colour calibration, thanks to the good agreement with the values obtained using the inspection chamber. The latter can therefore be replaced by the app, which allows reliable results to be obtained even when used on its own.

## 1. Introduction

In an increasingly competitive global agri-food market, consumer purchasing behaviour is often dictated by specific physiological and psychological triggers. These responses serve as cognitive shortcuts for evaluating product quality, bypassing a deeper understanding of complex production dynamics and intrinsic product properties. Extensive literature demonstrates that colour is the predominant driver of consumer attraction; specifically, saturated and vivid hues are perceived as more appealing [1,2,3]. For citrus fruits such as oranges and mandarins, an intense yellow-orange colour profile is universally associated with ripeness and optimal flavour, and is directly correlated with higher consumer acceptance. For citrus fruits typically eaten fresh, an excessively green hue can be perceived as a sign of immaturity and over-acidity, thereby deterring purchase. This visual influence is so powerful that degreening treatments, the ethylene-induced removal of chlorophyll, are standard industry practice to align product appearance with market expectations [4].

However, the context becomes more complex regarding specific citrus cultivars such as Bergamot (*Citrus bergamia*, Risso et Poiteau). This evergreen plant is primarily cultivated within a narrow 150 km coastal strip in the Province of Reggio Calabria (Southern Italy), where its essential oil is protected under the Protected Designation of Origin (PDO) “Bergamotto di Reggio Calabria” [5,6]. The fruit’s industrial applications are strictly dictated by its ripening stage: Yellow-ripe fruits are harvested for essential oil extraction (EO) and flavouring, whereas the unripe green fruits are sought after by the confectionery and perfumery sectors. Small, immature, grey-green fruits, often sun-scorched and prematurely dropped, are utilised for liqueur. Determining the ideal harvest window to maximise EO recovery has relied on the visual inspection of fruit pigmentation. Although the industry can still rely on the vast experience of growers, such manual assessments remain inherently subjective to individual bias. To achieve greater precision, modern agricultural practices are shifting toward digital image analysis. Up to now, colourimeters have been the usual instrument for colour-based maturity assessment because they are easy to use and provide accurate colour measurements. However, they are expensive and only measure a small area of the fruit’s surface [7], which may not be representative of the entire surface, particularly when the fruit is not uniform in colour. The technological shift towards smarter systems allows for an objective evaluation of fruit colour, providing data-driven insights to pinpoint the most effective harvesting moment. Researchers have explored various field-based sensing technologies, including thermal, multi- and hyperspectral sensors for chemical and physiological monitoring, stereo and colour cameras for spatial and volumetric data [8,9,10,11,12]. The challenge, therefore, also lies in capturing accurate data directly within the field environment [13]. In this context, modern smartphones have emerged as versatile and affordable tools, equipped with high-resolution cameras and robust computing power [14]. Their primary advantage lies in accessibility; practically all farmers possess these devices. Consequently, there is a growing trend towards developing dedicated field applications for such hardware [15]. As reported by [16], researchers in agricultural fields generally rely on calibration boards with patches of known colour values to ensure accuracy.

Recent research in smartphone colourimetry has focused heavily on mitigating device and environmental variability. Ref. [17] conducted a study in which a smartphone equipped with an RGB camera and a time-of-flight depth sensor was used for automated yield estimation of Chardonnay grapes. In this field acquisition context, characterised by uncontrolled lighting conditions and occlusions within grape clusters, a technique was developed to automatically identify grape berries in depth maps, exploiting distortion peaks caused by light diffusion within individual berries. Through a comprehensive scientometric review, ref. [18] analyses the rapidly growing body of literature on smartphone-based soil characterisation. The review identifies major challenges such as illumination variability and device-dependent differences, which continue to limit real-world applicability. On this topic, e.g., ref. [19] investigated the use of a colour reference plate to improve the accuracy of smartphone-based soil colour measurements under varying lighting conditions and across different devices. Their results confirm that raw colour measurements are strongly affected by both illumination and smartphone type, introducing significant bias. The use of colour calibration effectively reduced measurement errors for reference targets and improved overall consistency across devices and lighting conditions. Although the correction did not always fully eliminate bias for soil samples and Munsell chips, it consistently reduced variability, thereby enhancing the precision and comparability of smartphone-based colourimetric measurements in field applications. Ref. [20] introduced HueDx, a mobile-based colour correction system tailored for quantitative, on-site smartphone diagnostics. Designed for direct field deployment, this mobile application actively counters unpredictable ambient lighting conditions by executing white-balancing, multivariate Gaussian distributions, and histogram regression via dynamic, non-linear lookup tables. By performing real-time calibration at the point of care, the system restores field-captured images to near-imperceptible levels of colour difference, neutralising variations in brightness and colour temperature. Similarly, ref. [21] developed SMP-CC, an Android app for image acquisition, colour correction, and colourimetric analysis. By utilising a custom colour card, its built-in algorithm minimises colour differences compared to a colourimeter, effectively mitigating interference from varying lighting conditions and different smartphone hardware. Similarly, ref. [22] proposed a cloud-connected framework based on the HP3-ICT mobile application to standardise colourimetric measurements across a wide range of smartphone devices, from high-end to ultra-budget models. Their approach transforms raw RGB data into standardised HSV and CIELAB colour spaces and employs ensemble machine-learning algorithms, including Random Forest and XGBoost, to compensate for device-dependent colour variations. The proposed framework achieved a reduction of up to 75% in cross-device chromatic variability, demonstrating the effectiveness of data-driven colour calibration strategies for mobile imaging applications. Building on this requirement for precision, the advanced imaging capabilities of modern mobile devices now enable the automation of these processes by translating calibrated visual data into quantitative metrics. This allows for the direct derivation of colour indices, such as the Citrus Colour Index (CCI), the industry standard for determining harvest maturity and post-harvest treatments [23,24].

To facilitate the measurement of this index across different varieties at various maturity stages, the Instituto Valenciano de Investigaciones Agrarias (IVIA) developed a set of cards simulating fruit colour and texture at different ripeness levels for oranges and mandarins [25]. Building upon this approach, ref. [26] took it a step further by developing a free Android application that uses image processing techniques through the device’s built-in camera to assess citrus maturity across multiple varieties and stages. Nowadays, there are numerous smartphone-based applications in agriculture [27,28,29,30,31], and a controlled lighting chamber is often required to capture and store images without being affected by external lighting conditions and shadows [13,32]. Generally, two techniques are employed: in the first, images are transmitted to external servers or the cloud for analysis, with the results subsequently returned to the device; in the second, processing occurs directly on the phone using image-processing algorithms. However, both approaches require users to capture and save photographs first and then initiate the analysis process separately. This results in a deferred workflow, necessitating the management of stored files and often involving complex interfaces that reduce their practicality in the field.

Another major limitation of these state-of-the-art approaches is their strict reliance on dedicated colour references or specialised calibration charts, which restrict user flexibility and demand meticulous manual positioning. To address these limitations, we introduce a seamless, integrated framework that eliminates the need for cumbersome external colour targets. By coupling ArUco marker detection with histogram matching, the proposed system unifies automatic geometric localisation and colour normalisation into a single, robust workflow, significantly enhancing both automation and user flexibility.

Building upon the methodology proposed by [29], which employs a low-cost portable inspection chamber for in-field EO estimation through deep learning, this study introduces a bespoke mobile application that eliminates the need for cumbersome dedicated hardware. By integrating a colour-calibration reference card with a histogram matching algorithm, the application digitally compensates for variations in direct light, standardising the fruit’s colour profile in real time to ensure high-fidelity data acquisition under diverse environmental conditions. Consequently, this solution systematically neutralises perturbations caused by fluctuating ambient light and compensates for the inherent chromatic disparities arising from the heterogeneous sensor specifications of modern smartphone cameras. By replacing physical hardware with digital calibration, this approach facilitates seamless in-field data collection; it eliminates the ergonomic and positioning challenges associated with traditional inspection chambers, allowing for rapid and high-throughput analysis.

## 2. Materials and Methods

### 2.1. Design of the Reference Card

**Figure 1:**
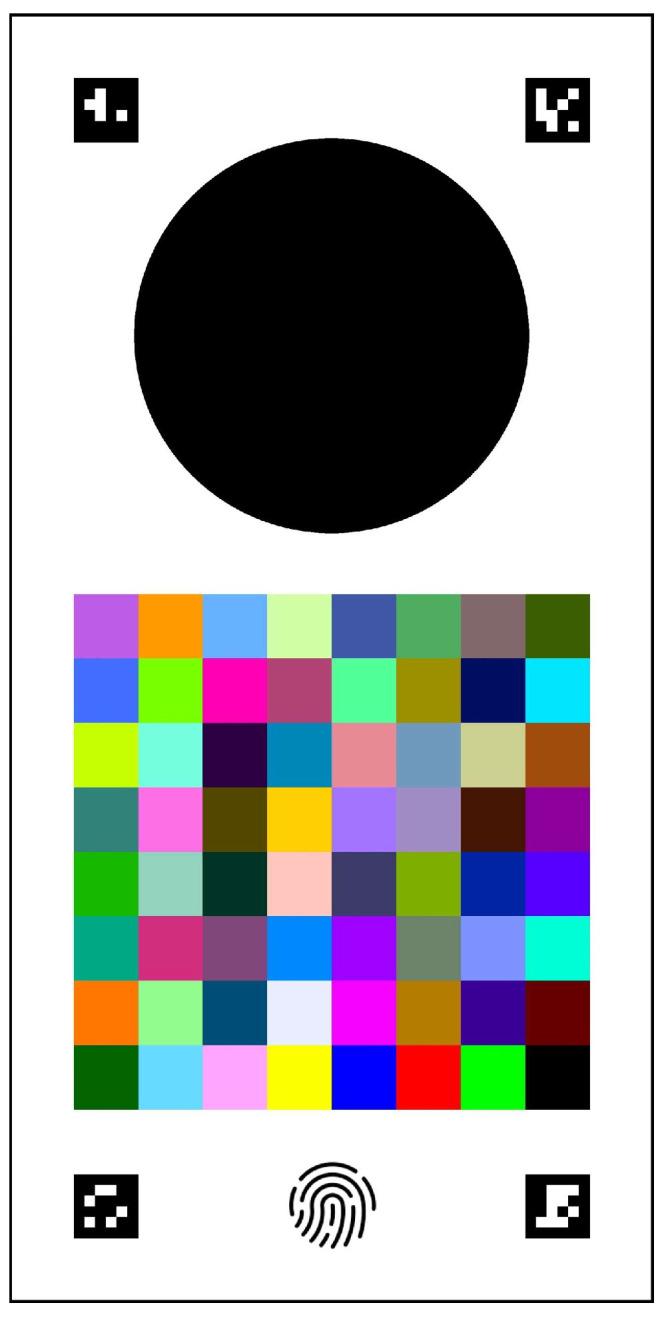
Colour reference card.

The first step of the methodology involved the development of a standardised reference card, specifically designed to automate the acquisition and calibration process (Figure 1). The card’s layout integrates several functional elements, most notably the chromatic matrix, with overall dimensions of 60 × 60 mm. This matrix is composed of an 8 × 8 RGB colour palette, which provides the essential profiles required for the light-compensation algorithms based on colour histogram matching. This technique is used to standardise the intensity distribution of an input image so that it matches that of a reference image. The operation relies on the cumulative distribution function (CDF): the CDF of the input image is mapped onto that of the reference image, transforming pixel intensity values to achieve statistical correspondence between the two images. Specifically, the CDF is computed for each channel of both the input and reference images; the input histogram is equalised, and each pixel is then mapped onto the reference CDF, adjusting its intensity to the corresponding reference value. This approach is particularly useful for correcting illumination variations, standardising colour across images acquired under different conditions, and preparing datasets for quantitative applications such as texture analysis, pattern recognition, or imaging instrument calibration. To ensure precise spatial identification, four ArUco Markers [33] were placed at the corners of the card, enabling the system to detect the object’s orientation with high accuracy and perform homographic rectification on the captured images. To neutralise spatial inconsistencies and perspective distortions resulting from manual acquisition, the images were subjected to a homographic transformation. This procedure corrected the scene geometry, ensuring that the projection of the samples remained constant and perpendicular to the lens plane throughout the entire dataset. Following this rectification, a two-stage geometric standardisation was performed. First, the images were adjusted to an aspect ratio of 2250 × 1000 pixels to ensure spatial symmetry. This proactive alignment facilitated a highly precise cropping operation to extract the primary Regions of Interest (ROIs): the colour card and the bergamot fruit surface. Although the high-resolution sensor captures significant morphological detail, this downsizing was performed to optimise the dataset, removing redundant high-frequency noise while preserving the essential chromatic and textural features of the flavedo. This approach ensures that the extracted areas are perfectly aligned, free from optical irregularities, and geometrically consistent. This provides a robust spatial framework to proceed with the colourimetric calibration. The design is further completed by a circular opening, which designates the specific area for the fruit image identification. To ensure the precision of the light-compensation algorithms, the 8 × 8 RGB colour palette was placed within a controlled inspection chamber, the same tool employed for in-field surveys in the reference study [29]. This stage was fundamental for acquiring a standardised reference card, which serves as the primary benchmark for the compensation logic. This calibration procedure, conducted under strictly controlled environmental conditions, enabled the establishment of a robust ground-truth benchmark by defining the baseline chromatic profiles of the matrix. By anchoring the digital data to these standardised measurements, the system ensures that the histogram matching logic remains accurate across variable ambient light conditions. Consequently, this reference is fundamental for validating the mobile application’s performance, ensuring that digital data acquired in the field remains consistent with the high-precision measurements obtained within the physical inspection chamber.

### 2.2. Algorithm for Colour Calibration and App Interface

**Figure 2:**
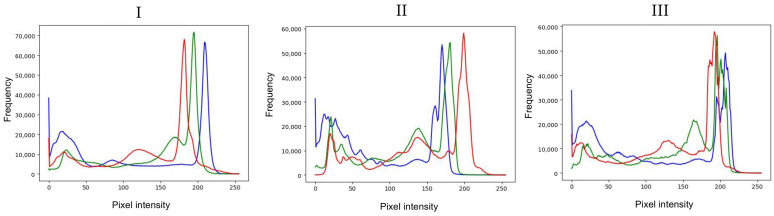
Colour card calibration histogram showing the main calibration workflow. The subfigures are described in the main text.

**Figure 3:**
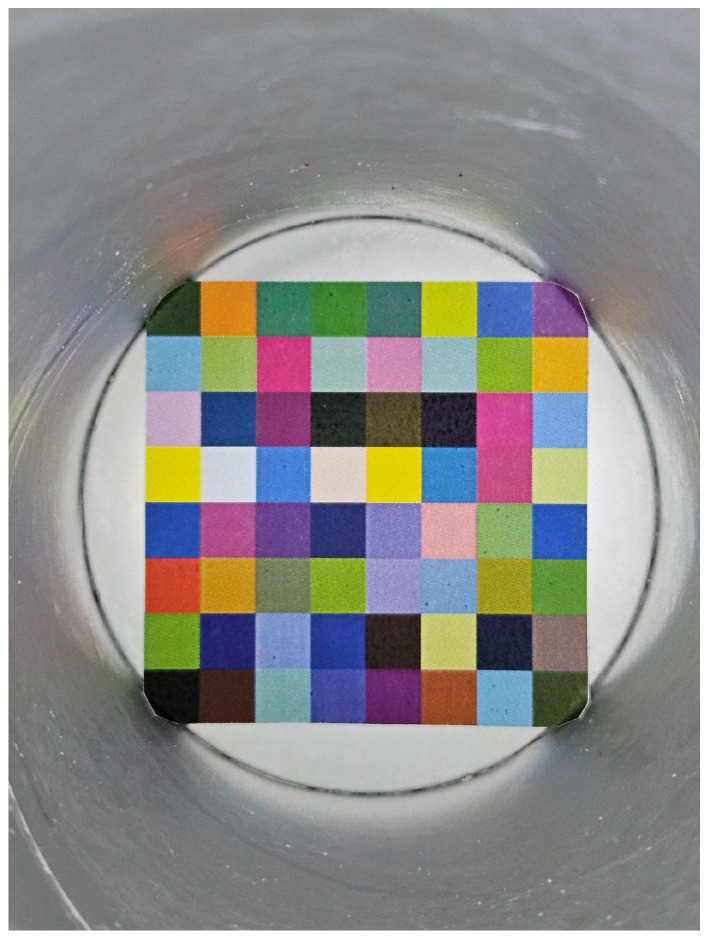
Colour card acquisition within the dark chamber.

The algorithm for computing the CCI value was implemented in an Android mobile application developed in Android Studio 2025 [34] as front-end, supported by a Flask 3.1-based backend. The acquired colour information was converted into the CIE L*a*b* colour space, as implemented in the open-source OpenCV 4.13 library [35]. This provides a perceptually uniform representation in which L* corresponds to lightness, while a* and b* describe the chromatic components along the green–red and blue–yellow axes, respectively. The transformation to CIE L*a*b* was performed to enable the computation of the CCI according to the procedure reported by [26]. This approach allows a more robust and consistent estimation of fruit colour, reducing the influence of illumination variability compared to RGB-based representations. During this phase, the acquired images and the calculated CCI values are transmitted to a server via REST API calls for subsequent analysis and evaluation. Figure 2 illustrates the main stages of the colour calibration algorithm: (I) starting from images of the reference card acquired inside the previously constructed dark chamber (Figure 3), as well as from those acquired in the open field, the RGB colour histogram is computed; (II) subsequently, the histogram matching algorithm determines the chromatic transformation required for the histogram of the image acquired outdoors to match that of the reference card inside the dark chamber; (III) this transformation is then applied to fruit images acquired outdoors. The histogram matching algorithm was implemented in Python using the pixel-ordering approach for exact histogram specification developed by [36].

The Android app’s interface is designed to facilitate colour measurement by the grower. When the application is running, it is sufficient to simply frame the superimposed colour card over the fruit and take the photo. The results of the processing appear on the screen, showing the CCI value and the photographed card, both with and without calibration. The CCI values exhibited significant fluctuations under different lighting conditions when processed without colour calibration. Conversely, the application of the proposed calibration algorithm consistently identified the true CCI value, effectively compensating for environmental variability and aligning the digital readings with the manual classification defined by the IVIA standard.

### 2.3. Description of the Tests

To validate the proposed approach, the full reference set of the citrus colour card developed by IVIA was employed [24,25]. This standardised scale comprises twelve distinct colour samples representing the entire ripening spectrum, corresponding to CCI values ranging from −24 to +22, also identified as card numerical values numbered from 1 to 12. Initially, all twelve colour cards were acquired in the dark room used in the previous study, to obtain stable reference measurements shielded from ambient light interference [23]. This setup was used only in the early development stage to reproduce controlled conditions for testing and debugging. For each card, six high-resolution images were acquired, resulting in a primary calibration dataset of 72 measurements (12 cards × 6 replicates). Data extraction was restricted to the central region of each image to minimise peripheral optical distortions.

Subsequently, the same twelve reference cards were acquired in an outdoor environment under three different lighting conditions to evaluate the system’s robustness to environmental variability. The first scenario (I) consisted of direct sunlight under a clear sky (5500 K). The second scenario (II) provided diffused natural illumination with minimal shadows (6000 K). The third scenario (III) corresponded to low-angle solar radiation at sunrise or sunset, characterised by warmer tones (3500 K). For each lighting condition, three images per card were acquired, yielding an additional dataset of 108 measurements (12 cards × 3 scenarios × 3 replicates).

Finally, the system was tested on bergamot citrus fruits (cv. Fantastico). A total of 338 fruits were manually classified using the IVIA citrus colour card to assign a reference ripeness level and subsequently labelled for tracking. Sampling was conducted randomly by selecting a variable number of healthy fruits throughout the orchard to ensure sample independence and minimise potential selection bias. The acquired data covered CCI values ranging from −12 to −4, corresponding to three ripeness classes (card numbers 4, 5, and 6), which represent the maturity stages most commonly encountered during commercial monitoring activities. All acquired samples were included in the analysis. The only excluded images were those in which the ArUco markers were not sufficiently visible or detectable, preventing the correct application of the colour calibration procedure. Since reliable colour calibration could not be guaranteed in these cases, they were considered invalid for the subsequent analysis. This three-step acquisition strategy ensured that the system calibration was first anchored to laboratory-grade reference values, then validated under varying natural illumination conditions, and ultimately tested on real samples, demonstrating its reliability and robustness in agricultural contexts.

**Figure 4:**
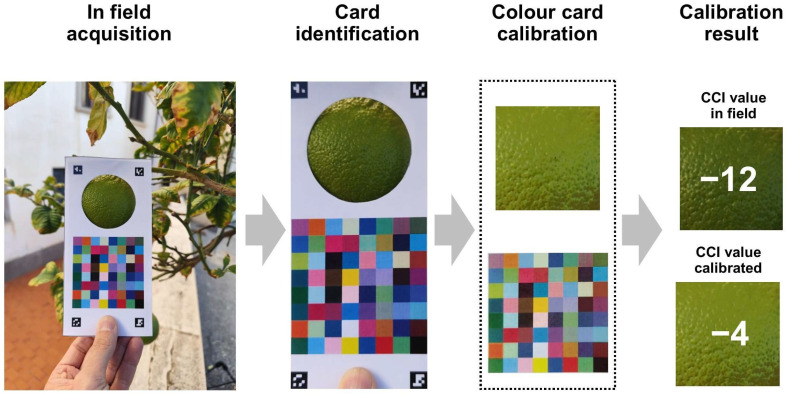
In-field colour standardisation methodology. The subfigures are described in the main text.

Data acquisition was performed using a smartphone Xiaomi Redmi Note 13 Pro smartphone (Xiaomi Corporation, Beijing, China) equipped with HyperOS, 12.0 + 6.0 GB RAM, and powered by a Snapdragon 7s Gen 2 octa-core Mobile Platform. The main camera sensor was a Samsung ISOCELL HP3 200 MP (Samsung Electronics Co., Ltd., Suwon, Republic of Korea). The smartphone’s automated configuration was utilised to leverage the Image Signal Processor (ISP) for dynamic exposure and focus adjustment in field conditions. All images were stored in high-quality JPEG format. Before processing, each image is resized to 3.15 MP (2048 × 1536 pixels) to reduce data transmission requirements, since the analysis is based on colour information rather than fine image details, high image resolutions are not required, making the application suitable even for older smartphones. Figure 4 delineates the computational pipeline designed for the chromatic standardisation of bergamot images acquired in situ via smartphone, a process fundamental to ensuring data consistency between field acquisitions and laboratory-trained predictive models. The procedure commences with the acquisition of the fruit images through the custom-made colour card featuring the circular aperture that exposes the epicarp (I); this ensures that both the reference colour matrix and the fruit are captured simultaneously under identical ambient lighting conditions. By utilising ArUco markers for robust spatial referencing, the system automatically isolates the card from the original scene to generate an 8 × 8 chromatic matrix (II), which represents the instantaneous lighting state. Concurrently, the software retrieves a “Colour Card Reference” from the database, corresponding to the matrix previously acquired within the controlled dark chamber used during the neural network training phase (III). The calibration algorithm then identifies the RGB channel discrepancies between the field-captured map and the reference, subsequently applying these transformational parameters to the fruit image to ensure optimal exposure (IV). This normalisation effectively replicates the effect of the dark chamber, eliminating environmental chromatic fluctuations, such as those illustrated in the transition from uncalibrated to calibrated CCI values, and enabling an accurate calculation of the CCI. For data processing, the entire methodology was implemented and analysed through purpose-developed code, encompassing the acquisition, chromatic calibration, and classification stages. In particular, the confusion matrix values were computed from the model outputs and subsequently visualised using the Matplotlib 3.9.10 library. Since illumination intensity was not directly measured during image acquisition, precise irradiance thresholds cannot be derived from the present dataset. Nevertheless, the acquisition process is subject to well-known operational limitations reported in several studies [37,38]. Smartphone-integrated camera modules present severe hardware and operational limitations because, unlike laboratory-grade computer vision systems, they are not originally designed for scientific purposes. The differences in smartphone camera hardware can lead to notable variations in colour detection and rendering in output data across different devices. In addition, delays in data transfer speed and storage may represent an important limitation, as this information is essential for the performance of statistical and mathematical algorithms used in image analysis. Consequently, they suffer from significant heterogeneity and fragmentation across different commercial brands, a lower native resolution, and a pronounced tendency toward increased digital noise when ISO sensitivity is raised [30,31,32,33,34,35,36,37,38,39,40].

The most critical operational challenge lies in the complete lack of standardisation and the strong dependence on ambient lighting conditions, which inevitably lead to underexposure in shadowed regions and overexposure caused by intense specular reflections. These exposure anomalies introduce significant visual noise, arbitrarily altering pixel values and removing genuine chromatic information before the image can even be processed. To overcome these limitations, the proposed methodology does not rely on portable imaging chambers to isolate the acquisition device from the external environment, nor on controlled, diffused LED illumination systems. Instead, it is based on transforming image intensities toward a reference distribution using histogram mapping techniques to improve the robustness and reliability of the analysis, even under uncontrolled acquisition conditions.

## 3. Results and Discussion

**Figure 5:**
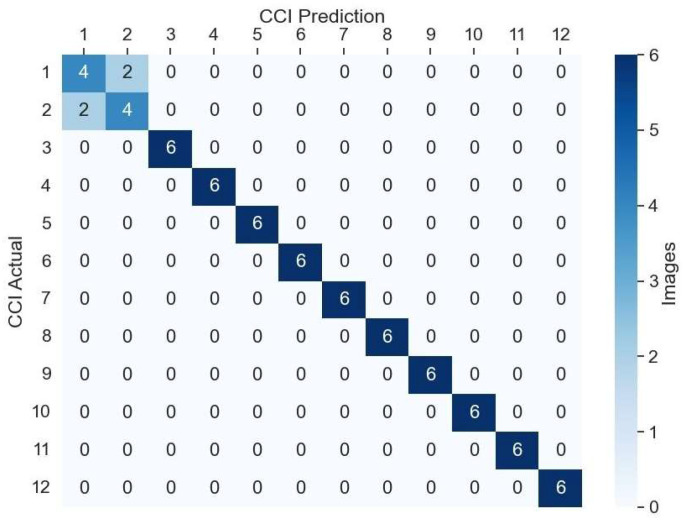
Confusion matrix of IVIA colour cards inside the inspection chamber.

Figure 5 shows the confusion matrix obtained for the CCI classification, directly validating the effectiveness of the proposed calibration strategy based on the IVIA citrus colour card acquisition within the inspection chamber setup, by card numerical values from 1 to 12. The samples along the main diagonal confirm that the normalisation procedure successfully compensates for environmental illumination variability, enabling highly accurate predictions. Notably, perfect classification is achieved for CCI classes from 3 to 12, demonstrating that the controlled acquisition of the reference colour card inside the inspection chamber provides a stable and repeatable chromatic baseline comparable to laboratory conditions. This highlights the robustness of the calibration pipeline, which transfers the controlled reference to field images through RGB correction. Slight misclassifications are observed only between classes 1 and 2, which can be attributed to the inherently low chromatic contrast at early ripening stages rather than to calibration limitations. These results confirm that the experimental setup effectively reproduces a standardised lighting condition, allowing the system to maintain high predictive performance even when applied to images acquired in uncontrolled environments.

**Figure 6:**
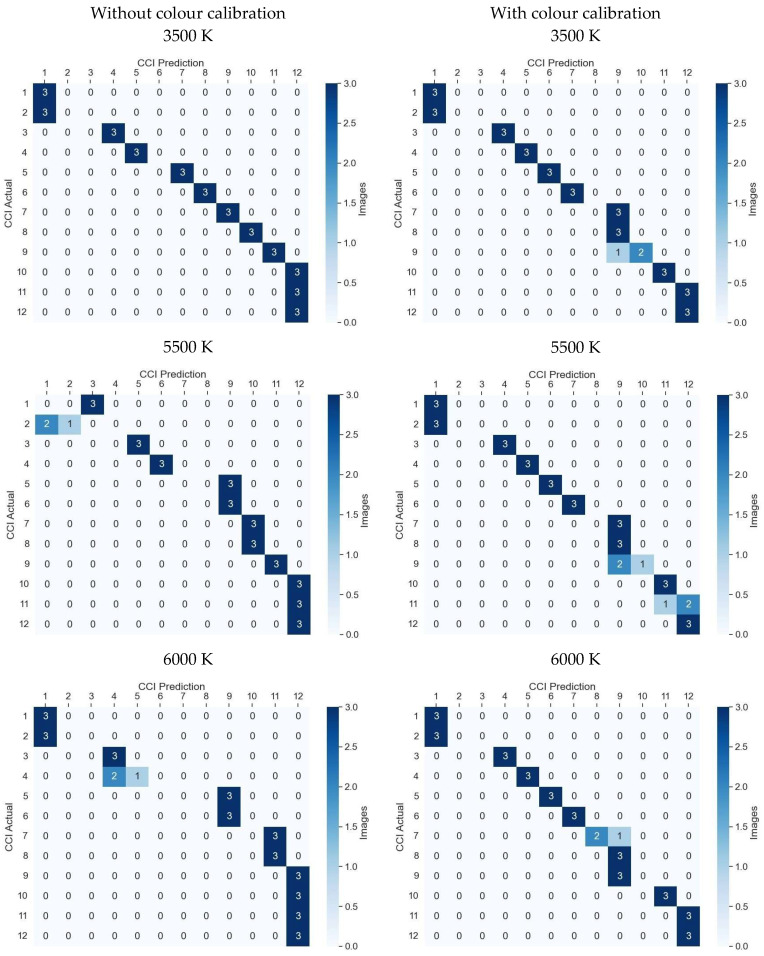
Confusion matrices of field tests under different illumination conditions, without (**left**) and with (**right**) colour calibration using the IVIA reference colour card.

Figure 6 presents the confusion matrices obtained from the same twelve reference cards under field conditions, comparing system performance without colour calibration (left panels) and with the proposed calibration procedure (right panels). In the absence of calibration, the predicted CCI values show a clear deviation from the ground-truth classes, with a noticeable dispersion of samples outside the main diagonal, particularly under non-uniform illumination. The chromatic calibration framework overall improves model performance compared to non-calibrated models, although some systematic errors persist across all illumination conditions. At 3500 K, global accuracy increases from 16.67% to 19.44%. In this condition, a strong overlap in the lower classes is still observed, with class 1 completely absorbing class 2 (100% recall for class 1 and 0% for class 2), resulting in 50% precision for class 1 and an F-score of zero for class 2. In the intermediate classes, a systematic +1 bias persists, eliminating true positives, while in the higher classes, only a partial recovery of the diagonal structure is observed. At 5500 K, accuracy improves from 11.11% to 25%. The same imbalance between class 1 and class 2 remains in the lower range, while in the intermediate classes, the +1 bias continues to affect precision and recall. However, compared to 3500 K, there is greater stabilisation of the distribution and a more evident recovery in the higher classes, with reduced saturation and better alignment with the diagonal. At 6000 K, accuracy rises from 22.22% to 25%, representing the best-performing condition among those analysed. Here, the model shows a clearer reduction of saturation effects in the higher classes and a more consistent recovery of the diagonal structure, while in the intermediate classes, the systematic +1 bias remains present but has a less significant impact on overall prediction coherence. The lower classes, however, maintain the same structural limitation. In summary, chromatic calibration progressively increases accuracy as colour temperature rises and improves the overall structure of predictions, particularly in the higher classes and at 6000 K. However, the presence of a systematic +1 offset in the intermediate classes and strong overlap in the lower classes requires a final adjustment of decision thresholds to translate structural improvements into real gains in precision, recall, and F-score. This behaviour may be related to the material properties of the IVIA colour cards. Since the cards are made of glossy laminated paper, specular reflections may occur during image acquisition, potentially affecting colour measurements and reducing calibration accuracy. Although controlled lighting conditions were adopted to minimise this effect, residual reflections may still contribute to colour estimation errors.

**Figure 7:**
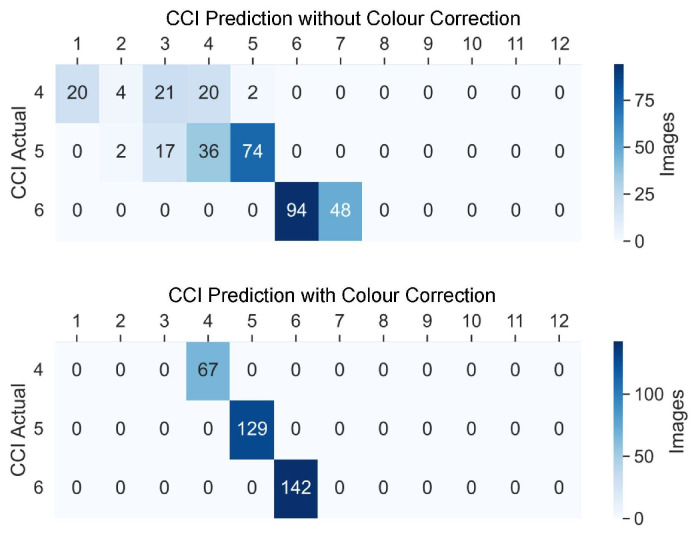
Confusion matrices of field tests on bergamot fruit, without (**up**) and with (**down**) colour calibration.

To evaluate the system performance under real operating conditions, 338 images of bergamot fruit were acquired from the bergamot fruits on the tree, for a total of 3 classes. The obtained results are reported in Figure 7, where the system behaviour is compared in the absence and presence of colour calibration based on the reference colour card. The quantitative evaluation confirmed the effectiveness of the proposed colour calibration framework. Without colour correction, the model achieved an accuracy of 55.62%, a recall of 55.62%, a precision of 86.25%, and an F1-score of 67.47%. After colour correction, all performance metrics increased to 100%, indicating that the calibration procedure successfully removed colour inconsistencies and enabled perfect classification of the CCI categories in the analysed dataset. In the absence of calibration, the estimated CCI values exhibit marked variability, with a significant dispersion of predictions from the ground-truth class. This instability can be attributed to environmental factors, particularly variations in illumination and acquisition angle, which affect the chromatic response of the image and compromise the reliability of the CCI computation. Conversely, when the colour calibration procedure is applied, the CCI values are stable and perfectly aligned with the ground-truth class for both analysed samples. This confirms that the method effectively compensates for environmental noise, ensuring robust, consistent, and accurate CCI estimation.

**Figure 8:**
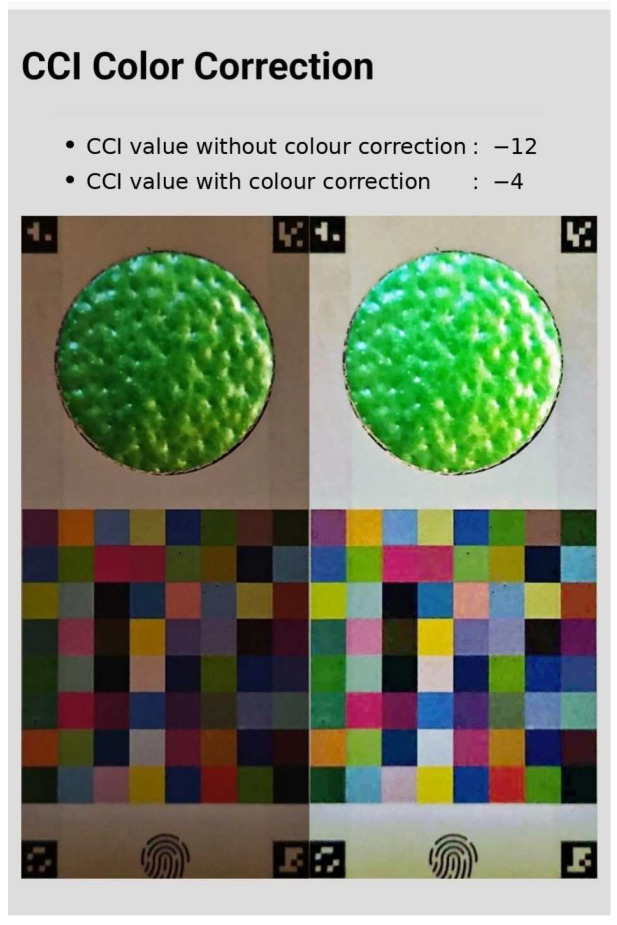
Application results of the mobile app.

Despite the success in field applications, the study reveals inherent limitations. Tests on IVIA reference cards under varying colour temperatures (3500 K–6000 K) highlighted residual systematic errors, such as a “+1” bias in intermediate ripening classes and difficulty distinguishing between early-stage classes. These limitations are likely linked to the glossy finish of the standard cards, which introduces specular reflections that can distort the underlying chromatic data even after compensation. Furthermore, the reliance on smartphone camera hardware introduces inevitable heterogeneity, which may affect the base raw signal quality. Figure 8 shows the mobile application interface, comparing its performance with and without colour calibration.

Recent studies highlight how smartphone applications are becoming increasingly valuable tools for rapid and accessible colour analysis across a wide range of contexts, from agriculture and food-quality monitoring to biomedical and healthcare applications. As reported by [23], a free Android-based application was developed for estimating the CCI directly from smartphone images using OpenCV image-processing techniques. The system calculates RGB values from selected fruit regions and converts them into the HunterLab colour space to estimate fruit maturity under both laboratory and field conditions. Tests performed on different citrus varieties and Android devices demonstrated good agreement with traditional colour evaluation methods.

A study conducted by [41] showed that the commercially available app Colour Grab can be used to monitor the degradation of reheated cooking oils through CIE Lab* colour analysis performed directly on smartphone-acquired images, highlighting the potential of low-cost mobile technologies for preliminary food screening applications. In the biomedical field, the reliability of smartphone-based mHealth systems is closely related to the accuracy of colour normalisation procedures. In this regard, DailyDerma was evaluated as a support tool for monitoring skin erythema associated with canine atopic dermatitis. 

The research carried out by [42] demonstrated that the integration of Colour Calibration Targets (CCTs), containing reference colour patches with predefined values, significantly enhances the consistency and reliability of smartphone images. Through the mapping between captured and reference colours, the acquired images can be converted into a standardised and device-independent colour space, thereby improving the robustness of smartphone-assisted biomedical assessments.

Another important aspect concerns colour visualisation consistency across mobile displays. Many smartphone applications and web platforms assume compliance with the device-independent sRGB colour space, despite the fact that accurate display characterisation generally requires specialised instrumentation and complex calibration workflows. To overcome this limitation, ref. [43] proposed a pseudo-device-independent framework for Apple mobile displays based on the Mobile Display Characterisation and Illumination Model (MDCIM). Their results demonstrated that a generalised calibration model can markedly improve colour reproduction accuracy on Apple devices without the need for individual device characterisation, thus increasing the reliability and practicality of smartphone-based colour visualisation systems in real-world scenarios.

This research establishes a scalable, low-cost methodology for precision agriculture. By eliminating the need for bulky portable imaging chambers or controlled LED environments, the framework relies on high-precision colour analysis. It provides a robust tool for growers, shifting from subjective visual inspection to objective, data-driven maturity monitoring that is device-independent and to fluctuating field lighting.

While the individual methodologies utilised in this framework, namely ArUco markers, homographic rectification, and RGB histogram matching, are well-established in the image processing literature, the integration of these discrete processes into a unified Android mobile application was achieved. By embedding this end-to-end computational pipeline directly onto a commercial smartphone framework, this research provides a real-time, robust analytical tool capable of mitigating severe environmental lighting fluctuations directly within the bergamot orchard. Consequently, the core innovation shifts from individual algorithmic development to the architectural and operational deployment of an accessible precision agriculture solution.

## 4. Conclusions

This study presented a novel mobile-based system for the automated assessment of fruit maturity, specifically designed for bergamot cultivation. The methodology involved a standardised reference card equipped with an 8 × 8 RGB chromatic matrix and ArUco markers for precise spatial homographic rectification. The work verified the system’s performance through a three-stage validation process: anchoring the calibration to controlled laboratory conditions, evaluating robustness across varying outdoor colour temperatures, and conducting field trials on bergamot fruits.

The results demonstrate that the proposed histogram-matching approach effectively mitigates illumination-induced chromatic shifts, yielding stable and accurate CCI values. The significance of this research lies in its ability to deliver laboratory-grade accuracy in a field setting using standard consumer smartphones.

By providing a reliable, user-friendly tool for real-time ripeness monitoring, this application facilitates informed decision-making in commercial orchard management, offering a sustainable path forward for non-destructive crop quality assessment.

## Data Availability

The data presented in this study are available on request from the corresponding author due to ongoing software validation and development activities associated with a mobile application that has not yet been publicly released.
